# Diffusion Model-Guided Inverse Design of Bimetallic Catalysts for Ammonia Decomposition

**DOI:** 10.1021/jacs.5c14652

**Published:** 2025-12-19

**Authors:** Jiaqi Yang, Kailong Ye, Shaohua Xie, Qiang Li, Charles Milhans, Fudong Liu, Fanglin Che

**Affiliations:** † Department of Chemical Engineering, 542588 Worcester Polytechnic Institute, Worcester, Massachusetts 01609, United States; ‡ Department of Chemical Engineering, 14710 University of Massachusetts Lowell, Lowell, Massachusetts 01854, United States; § Department of Chemical and Environmental Engineering, Bourns College of Engineering, Center for Environmental Research and Technology (CE-CERT), Materials Science and Engineering (MSE) Program, UCR Center for Catalysis, 8790 University of California, Riverside, Riverside, California 92521, United States

## Abstract

In the past decade, artificial intelligence and deep learning have played increasingly prominent roles in materials design and discovery. Among these, generative AI models, known for their ability to create unique and complex structures, have emerged as state-of-the-art tools for materials screening due to their high efficiency and low computational cost. In catalysis, one of the major challenges is identifying promising material candidates within an immense chemical space. This challenge can be addressed using generative approaches, such as diffusion-based inverse design models. In this study, we present a machine learning-guided workflow that employed a diffusion model for the inverse design of bimetallic alloy catalysts for low-carbon ammonia decomposition, a key reaction for ammonia emission control and sustainable hydrogen production. Catalyst candidates were evaluated using nitrogen adsorption energy as the key descriptor, inspired by multiscale modeling. The proposed workflow identified low-cost, environmentally friendly catalysts with excellent catalytic performance, which have been validated theoretically and experimentally. Our framework decoupled the generative and property-prediction components, enhancing both flexibility and accuracy in the catalytic material design process.

## Graphical abstract

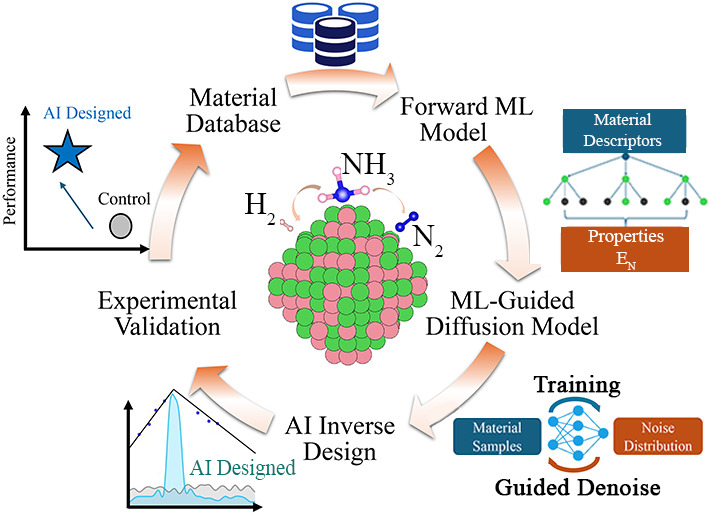

## Introduction

Maritime shipping accounts for approximately 3% of global CO_2_ emissions (∼1 billion metric tons).^1^ To reduce emissions, a *Nature Energy* analysis^2^ compared alternative marine fuels, including hydrogen, ammonia, methane, and methanol, and identified ammonia as the most balanced carbon-free fuel for mobile application due to its economic viability, high energy density, and ease of storage and transport.^3,4^ Although NH_3_ combustion can be retrofitted into existing engines, it faces challenges such as low efficiency (30–40%), NO_*x*_ emissions, slow flame speeds, narrow flammability limits, a high autoignition temperature, and combustion instability.^5–8^ Onboard NH_3_ decomposition to produce H_2_, coupled with proton exchange membrane fuel cells (PEM-FCs),^9,10^ can achieve a higher efficiency (50–60%) with zero NO_*x*_ emissions.^11^ However, this approach requires high-temperature ammonia decomposition (600–800 °C) over earth-rare Ru catalysts and potential H_2_ purification.^12–15^ While NH_3_ combustion is a short-term fix, NH_3_ decomposition with PEM-FCs offers a sustainable path for maritime transportation. At the same time, NH_3_ decomposition at low concentrations represents a potentially important reaction for emission control when NH_3_ acts as a pollutant.

To overcome the high-temperature challenge and enable ammonia decomposition using earth-abundant, noncritical metal catalysts, alloys rich in transition metals offer strong catalytic potential due to their tunable electronic structures and geometric configurations, which contribute to enhanced catalytic activity.^16,17^ These alloy catalysts (such as CoMoFeNiCu) have demonstrated enhanced catalytic performance for NH_3_ decomposition, with improvement factors exceeding 20 times over that of Ru catalysts via lowering the energetics of N–H bond activation and N≡N bond formation during NH_3_ decomposition.^18,19^ H_2_ formation is considered a non-rate limiting step and less dominant during NH_3_ decomposition.^20^ Therefore, optimizing alloy catalysts (such as components and ratios) has the potential to enhance reaction rates, achieve complete conversion at lower temperatures, improve the energy efficiency of ammonia decomposition, and reduce hydrogen production costs.

Building on the promising performance of these alloys, our previous work^21^ employed multiscale simulations integrating density functional theory (DFT) and microkinetic modeling (MKM) to identify key descriptors, such as the binding energy of key intermediates, that govern catalytic activity. The study demonstrated that N≡N bond formation is the rate-determining step, controlled by a balance between nitrogen surface coverage and activation barriers. A volcano-shaped relationship between the catalytic activity (TOF) and adsorption energy of key intermediate nitrogen (*E*_N_) was observed over the flat surfaces of transition metals, with the maximum activity occurring at an optimal *E*_N_ of approximately −0.9 eV, close to the nitrogen binding energy on Ru(0001). Catalysts with strong nitrogen binding hinder N≡N bond formation due to the high activation barriers, while those with weak binding fail to maintain sufficient N* coverage. This clear quantitative trend establishes *E*_N_ as a robust and physically meaningful descriptor for predicting the catalytic performance in ammonia decomposition.

Having established a quantitative descriptor for catalytic activity, the next challenge lies in efficiently navigating the immense compositional space of alloy catalysts to identify the optimal formulations. Traditional trial-and-error approaches, whether experimental or high-throughput computational, are time- and resource-intensive. To efficiently navigate this complexity and identify optimal alloy catalysts for ammonia decomposition, we leverage generative modeling techniques, which can rapidly predict catalytic performance based on a limited training database and guide the inverse design of high-performing alloy catalysts.

Recent advances in generative modeling have introduced powerful new tools for data-driven materials discovery and design. Among these, diffusion models have gained significant attention for their ability to generate high-fidelity, diverse, and controllable data across various domains.^22^ Initially introduced as an alternative to generative adversarial networks (GANs) and variational autoencoders (VAEs), diffusion models offer several advantages, including stable training dynamics, high-quality sample generation, and strong theoretical grounding.^23,24^

Mechanistically, diffusion models are operated by simulating a stochastic process that gradually corrupts data with noise and then learning to reverse this process to recover the original distribution.^23,25^ This forward–reverse diffusion framework, often modeled using a Markov chain and parametrized by a neural network, enables the generation of complex, structured outputs from random noise.^26^ Originating from principles in nonequilibrium thermodynamics, this mechanism makes diffusion models particularly well-suited for capturing the intricate patterns and constraints found in materials systems, such as alloy design.^27^

In materials science and catalysis, generative AI models, particularly diffusion models, are emerging as transformative tools for exploring complex material spaces under defined conditions. These models have demonstrated remarkable success in applications ranging from biomolecular modeling and drug design^28^ to three-dimensional structure^22^ prediction and conditional generation.^29^ Recent advances in diffusion and flow-matching architectures have further enhanced their efficiency and accuracy, positioning them as highly promising frameworks for inverse design and property-driven materials discovery.^22,25,26,29,30^

In recent decades, diffusion models have been widely devoted to materials and chemistry design. Weiss et al. created a guided diffusion model (GaUDI) for molecular inverse design, which generated novel desired molecules with both point-wise targets and open-ended targets.^31^ Zeni et al. constructed MatterGen, a generative model that generated stable, diverse inorganic materials with property constraints, including chemistry requirement, symmetry, mechanical, electronic, and magnetic properties.^29^ Joshi et al. introduced the All-atom Diffusion Transformer (ADiT) to the field.^27^ The unified latent diffusion framework can generate both novel molecules and periodic materials by translating structures into a latent space using a pretrained autoencoder. However, applying diffusion models for inverse design targeting catalytic performance remains uncommon in catalyst development.

To address this, we present our integrated diffusion model workflow for the design of bimetallic catalysts, specifically aimed at targeted adsorption energies and catalytic performance. This workflow combines a predictive machine learning (ML) model with a generative diffusion model to efficiently explore the design space of low-cost, high-performance bimetallic alloy catalysts for ammonia decomposition. By combining the forward ML model and the generative diffusion model, a highly efficient and low computational expense workflow is generated toward catalyst design.

The diffusion model, with strong generative capability, enables broad and efficient exploration of the materials design space.^23,24^ Other generative approaches, such as the global optimization method, primarily target a single optimal solution. While global optimization excels at exploitation, it often becomes trapped in local minima and overlooks diverse candidates with comparable or superior potential.^24,32^ The diffusion model, by learning the underlying probability distribution of structural and compositional data, can generate diverse, property-guided catalyst candidates that capture both composition and adsorption site information, features essential for accurately describing complex catalytic systems.^23^

In this work, we construct a descriptor matrix that encodes the metal composition, bulk structural features, and adsorption site characteristics. Because a single structure may contain multiple distinct adsorption sites, the diffusion model provides a natural framework for producing both compositional and site-specific outputs, enabling direct structural reconstruction and subsequent DFT validation. Its versatility and scalability also allow straightforward extension to high-dimensional and complex systems such as high-entropy alloys, where traditional optimization methods become computationally prohibitive. Starting with bimetallic alloys as a tractable test case, we aim to refine and extend this diffusion-based framework toward increasingly complex catalytic systems, where its generative flexibility offers a powerful and efficient route for accelerated catalyst discovery.

By applying the diffusion model workflow, we successfully decrease approximately 90% of required DFT simulations and millions of CPU hours compared with traditional high-throughput DFT screening. We present a detailed framework for implementing and evaluating diffusion models in the context of catalyst design, highlighting the potential of generative approaches to accelerate the discovery of novel catalytic materials.

## Results

### Inverse Design Workflow

The inverse design workflow incorporates two pretrained machine learning (ML) models for designing bimetallic alloys. The first is a generative diffusion model, trained to produce unconditional samples from a given data distribution. The second is a predictive model trained to estimate nitrogen (*E*_N_) adsorption energy, a key descriptor of catalytic performance identified in our prior multiscale modeling of ammonia decomposition.^21^ The predictive model serves both as a guiding function and as an evaluation method for generating new samples by using the diffusion model. The detailed workflow is illustrated in Figure 1.^18^

**Figure 1:**
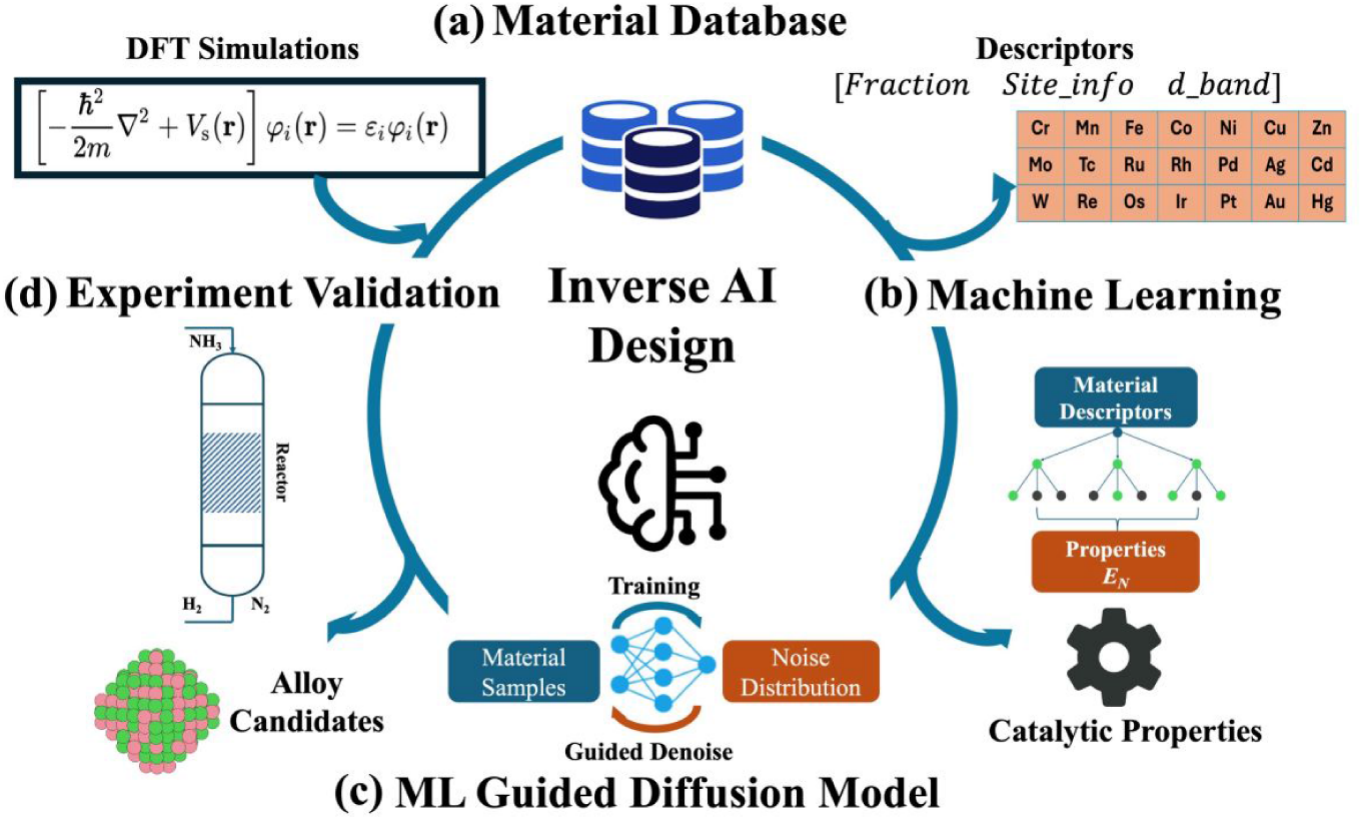
Workflow illustration of machine learning (ML)-guided inverse design of bimetallic alloys for NH_3_ decomposition. (a) DFT-generated materials database containing optimized structures, nitrogen adsorption energies (*E*_N_), and *d*-band characteristics; (b) forward ML model based on a Random Forest algorithm for predicting nitrogen adsorption energies across different adsorption sites and alloy surfaces; (c) ML-guided diffusion model for generating promising bimetallic alloy candidates; and (d) DFT and experimental validation of the diffusion model-generated alloys.

The training data for both models are generated and optimized by using density functional theory (DFT) simulations. The chemical space spans 15 late transition metals from the 3d (Fe–Cu), 4d (Mo–Ag), and 5d (W–Au) series. We focused on face-centered cubic structures with compositions of A1B0, A3B1, A7B1, and A15B1 (Figure S1). The resulting database includes N adsorption data for 3450 inequivalent 3-fold hollow sites (fcc and hcp) on the (111) surfaces of these alloys, which were used to train the models.

Surface segregation introduces an additional layer of complexity in the alloy systems. However, the hollow sites formed on the (111) surface upon segregation coincide with those in our compositional models (Figure S2), ensuring consistency across different alloy configurations. Thus, the effects of segregation are inherently represented by varying alloy compositions and ratios, and this factor was explicitly examined only for AI-predicted models.

To narrow the chemical space arising from catalyst morphology and surface coverage, we further (i) assessed the descriptor *E*_N_ for (211) step sites via microkinetic modeling and (ii) evaluated *E*_N_ across a range of coverages on Ni–Mo alloys. As shown in Figure S3, both terrace and step surfaces display a consistent volcano-type correlation between the catalytic activity and *E*_N_. Moreover, *E*_N_ follows the same trend with increasing coverage across various Ni–Mo compositions (Figure S4). Consequently, the (111) surface with low surface coverage of nitrogen suffices to capture alloy-wide trends. Additional simulation details are provided in the (Computational Details SI).

The diffusion model samples from a tractable noise distribution and iteratively denoises the signal, following the standard diffusion sampling approach. In this work, we first constructed a bimetallic alloy database with randomly sampled alloy species (Figure 1a). The fractions of metal compounds are generated with the following ratios: 1:1, 3:1, 7:1, and 15:1. The constructed bimetallic structures are modeled for N adsorption on various sites using DFT simulations, capturing structures, N adsorption energies, and *d-*band properties (Figure 1b). Each bimetallic alloy is encoded into a matrix descriptor that includes the composition, site-specific information, atomic properties, and *d-*band features (Table 1).

**Table 1:** Descriptor Details of the Elemental Composition, Atomic Properties, *d*-Band Filling, and Adsorption Site Characteristics

Composition	Elemental Properties	Site Information	Electronic Properties
Fraction of Metal A	Atomic Radius	Hollow Site Type (fcc/hcp)	*d*-band filling of 1st nearest neighbor
Fraction of Metal B	Atomic Spatial Extent	1st nearest neighbor fraction	Average *d*-band filling of 2nd nearest neighbor
	Ionization Energy	2nd nearest neighbor fraction	
	Electron Affinity		
	Electron Negativity		

To train the diffusion model, Gaussian noise is added to each sample over 25 iterations. A maximum perturbation limit of 5% is imposed on each iteration to ensure that the perturbed samples remain close to the original sample distribution (Figure 1c). Because the generative model must operate within the physically meaningful chemical space, several physical constraints were embedded into the model’s loss function to enforce realistic alloy generation: (1) the total number of nonzero elemental fractions was limited to two; (2) the *d*-band filling for each noisy iteration was restricted to values below 1; (3) key atomic properties were confined to defined ranges (e.g., *d*-band filling within [0.7 e, 1 e] and atomic radius within [1.3 Å, 1.7 Å]). These constraints were implemented via ReLU-based penalty functions, which substantially increase the loss when any generated configuration violates the specified conditions.

Additionally, the intermediate outputs of the generative model are evaluated by the predictive model, which estimates the N adsorption energy. The gradients of *E*_N_ are used as a guiding signal during the inverse design process by introducing a correction term into the loss function (Figure 1d). This strategy enables the diffusion model to generate bimetallic alloys with adsorption energies close to the target value (*E*_N_ ≈ −0.9 eV), based on our previous multiscale simulation findings.

The remapping from a descriptor matrix to DFT-ready structures, shown in Table 1, converts the local adsorption environment in terms of site type and its first-nearest neighbors. The generation of catalyst candidates is guided by the following physically grounded assumptions:

(1) The alloys exhibit minimal segregation.
(2) The exposed alloy surface is restricted to the (111) facet, consistent with both our multiscale simulation-based volcano analysis (Figures S3 and S5 in the Supporting Information) and the training database, which are constructed on this facet. Notably, we also examined stepped surfaces such as the (211) facet, which produced a volcano trend similar to that of the (111) surface.
(3) The local adsorption-site environment is uniquely determined by the first nearest neighbors (Figure S6).

Under these assumptions, the reconstruction procedure can yield multiple alloy configurations that share identical local adsorption sites. Variations in atomic configurations arise only beyond the first-nearest neighbors, which are not expected to significantly influence adsorption energetics for this alloy system (Figures S7–S9).^27,33^ Recent studies further reinforce this assumption. Ulissi and coworkers^33^ recently adapted and fine-tuned the Open Catalyst Project (OC20)-trained EquiformerV2 for high-entropy alloys (HEAs), demonstrating that explicitly conditioning on the local binding-site environment, i.e., the atoms directly constituting the site, enables accurate adsorption-energy predictions of reaction intermediates. Dean et al. introduced a universal adsorption model that integrates DFT with machine learning, identifying local coordination and neighboring-atom identity as the dominant factors controlling adsorption across diverse nanoparticles and compositions.^34^ Similarly, Li et al. created AutoML to determine the key features controlling adsorption energies on binary alloy surfaces. The AutoML model concludes that local adsorption site geometric descriptors, such as coordination and local arrangement of neighbors, are the primary physical determinants of adsorption energies, with electronic descriptors playing a secondary role.^35^

### Forward Machine Learning Model for Predicting N Adsorption Energy

The predictive ML model plays a vital role in guiding the inverse design process by steering candidate materials toward optimal catalytic performance. For ammonia decomposition, the nitrogen adsorption energy (*E*_N_) is a key descriptor of catalytic activity. This relationship is captured in a volcano plot (Figure S5),^7^ derived from microkinetic modeling and DFT simulations. The plot indicates peak catalytic performance at *E*_N_ of approximately −0.9 eV, which corresponds closely to the activity maximum near Ru-based catalysts.^21^

To efficiently screen bimetallic alloys as potential alternatives to near-peak earth-rare Ru catalysts, we developed a forward regression model capable of rapidly and accurately predicting E_*N*_, enabling a high-throughput evaluation of candidate structures, as illustrated in Figure 1b. The machine learning model was trained on a data set of DFT-simulated bimetallic alloy samples, with each sample described by features such as elemental composition, atomic properties, *d*-band filling, and adsorption site characteristics (Table 1). Among the regression methods tested, the Random Forest algorithm yielded the highest predictive accuracy. As shown in Figure 2b, the model achieved a mean absolute error (MAE) of 0.12 eV in predicting the nitrogen adsorption energy.

**Figure 2:**
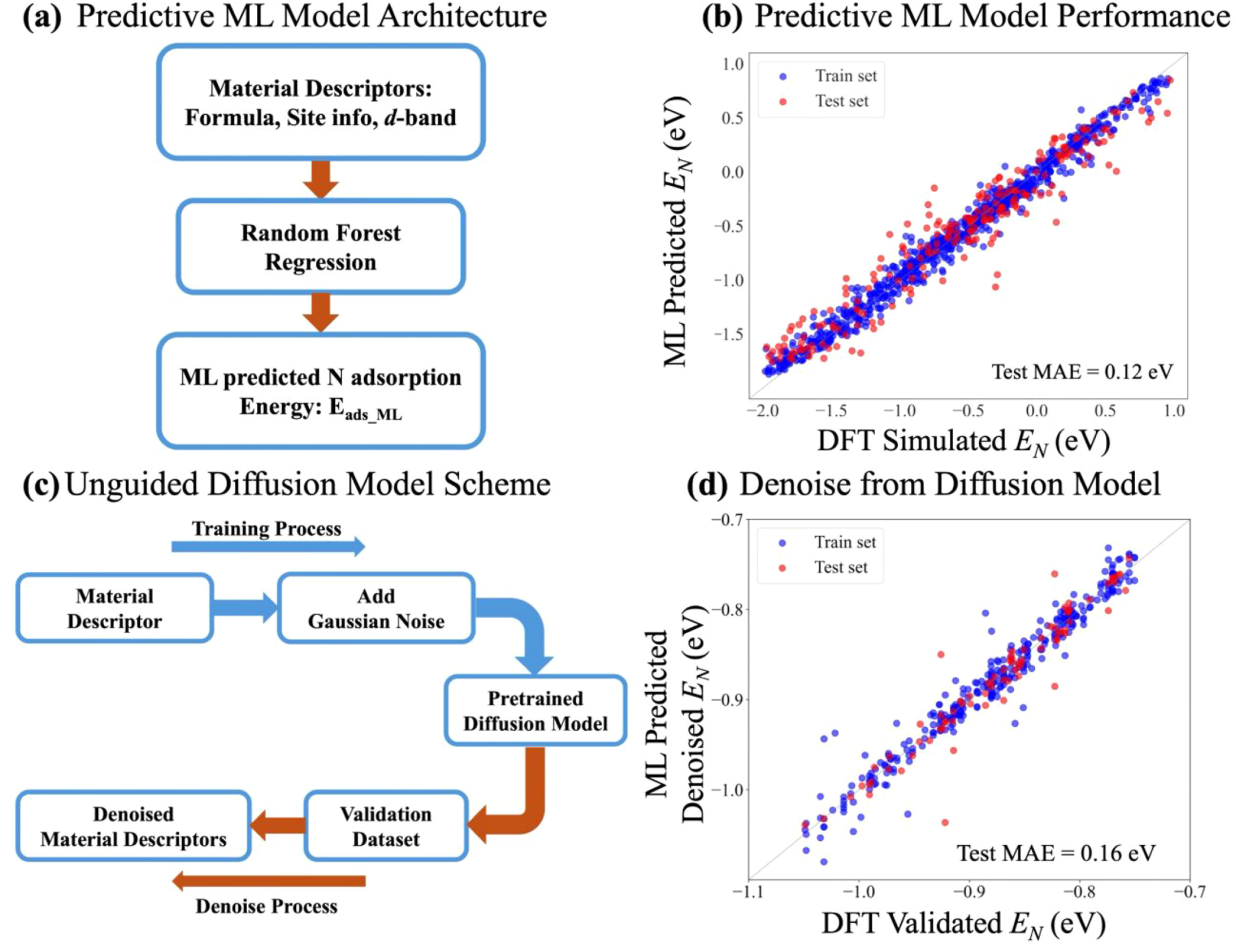
Performance of the predictive ML model and denoising capability of the diffusion model without a guidance function. (a) Schematic of the forward ML model used to predict nitrogen adsorption energies; (b) accuracy comparison between ML-predicted and DFT-validated nitrogen adsorption energies; (c) schematic of the diffusion model without a guidance function; (d) accuracy comparison of the unguided diffusion model’s denoising reconstruction, evaluated using ML-predicted and DFT-validated nitrogen adsorption energies.

To interpret the model, we performed SHapley Additive exPlanations (SHAP)^36^ analysis (Figure S6). The analysis identified *d*-band filling as the most influential factor, which is consistent with established principles in previously reported literature.^21^

### Unguided Bimetallic Alloy Design

Before introducing the guided inverse design process, we first evaluated the intrinsic generative capability of the diffusion model to produce physically valid and diverse bimetallic alloy configurations. The model is trained on a DFT-simulated database comprising bimetallic alloys made from 19 different elements across various compositional ratios. A total of 2,500 unique samples were selected within the nitrogen adsorption energy range of −2 to 1 eV.

Each alloy sample was represented by a descriptor matrix encoding elemental fractions, adsorption site information, and electronic structure features such as *d*-band filling (Table 1). To introduce controlled stochastic variability, trackable Gaussian noise was added to each sample with 25 iterations to make training noised arrays,^22,28^ with a maximum perturbation of 5% per iteration to ensure samples remained close to the original data distribution. Repeating this process for each of the 2,500 base samples generated an ensemble of noisy-clean data pairs, providing a comprehensive and diverse training set. This data set enabled the diffusion model to learn the underlying data distribution and develop the capacity to reconstruct denoised representations from ambiguous or incomplete inputs.

The diffusion model was trained to learn the noise patterns between these noised matrices by minimizing the discrepancy between the predicted and actual noises added during the perturbation process. The training objective is formalized through the loss function shown in eq 1,^25^

1 $$L_{simple,t}=E_{x0∼q;z∼N\left(0,1\right)}\left[{||\epsilon_{\theta }\left(x_{t},t\right)-z||}^{2}\right]$$

Where *ϵ*_*θ*_(*x*_t_,*t*) is the model output noise vector and *z* is the trackable Gaussian noise. *E*_*x*0*∼q;z∼N*(0,1)_ is the expectation over integrals of term ||*ϵ*_θ_(*x*_t_,*t*) – *z*||^2^.

To evaluate the accuracy of the diffusion model, we compare the denoised samples generated by the model with the corresponding DFT-calculated values from the original data set. The denoised samples are assessed using the predictive ML model to estimate their nitrogen adsorption energy. Ideally, the difference between the predicted *E*_N_ values of the denoised samples and the original DFT values should be minimal. As shown in the parity plot in Figure 2d, the MAE between the denoised and DFT values is 0.16 eV, demonstrating the model’s ability to generate physically meaningful and accurate candidate structures that capture the underlying data distribution.

### ML-Guided Bimetallic Alloy Design

To bias the generative process toward bimetallic alloy structures with desired catalytic properties, we introduce a guiding function layer into the diffusion model architecture. This layer integrates a pretrained predictive ML model for N adsorption energy, which serves as the evaluation during each denoising step.

At each iteration, the gradient of *E*_N_ between successive iterations is computed to assess whether the generation trajectory is converging toward the target adsorption energy of −0.9 eV, a value associated with near-optimal catalytic activity for ammonia decomposition (Figure S5). If the *E*_N_ gradient indicates movement toward this target, the intermediate sample is retained; otherwise, a penalty is applied to the loss function to disincentivize nonoptimal directions. The penalty is modulated using a rectified linear unit (ReLu) function to selectively amplify deviations from the desired *E*_N_ gradient.

To enhance computational efficiency and sample quality, the denoising process is terminated for any candidate that accrues more than five successive interactions due to nonconvergent *E*_N_ gradients, under the assumption that such samples are unlikely to achieve the desired adsorption characteristics.

The effectiveness of the guiding function is illustrated in Figure 3b, where the gray histogram represents the *E*_N_ distribution of the initial input samples, and the blue histogram shows the distribution after guided denoising. The postguidance distribution demonstrates a pronounced shift, with most samples concentrated in the range of −1.0 eV to −0.8 eV, confirming that the guiding function effectively directs the generative process toward high-performance catalytic candidates.

**Figure 3:**
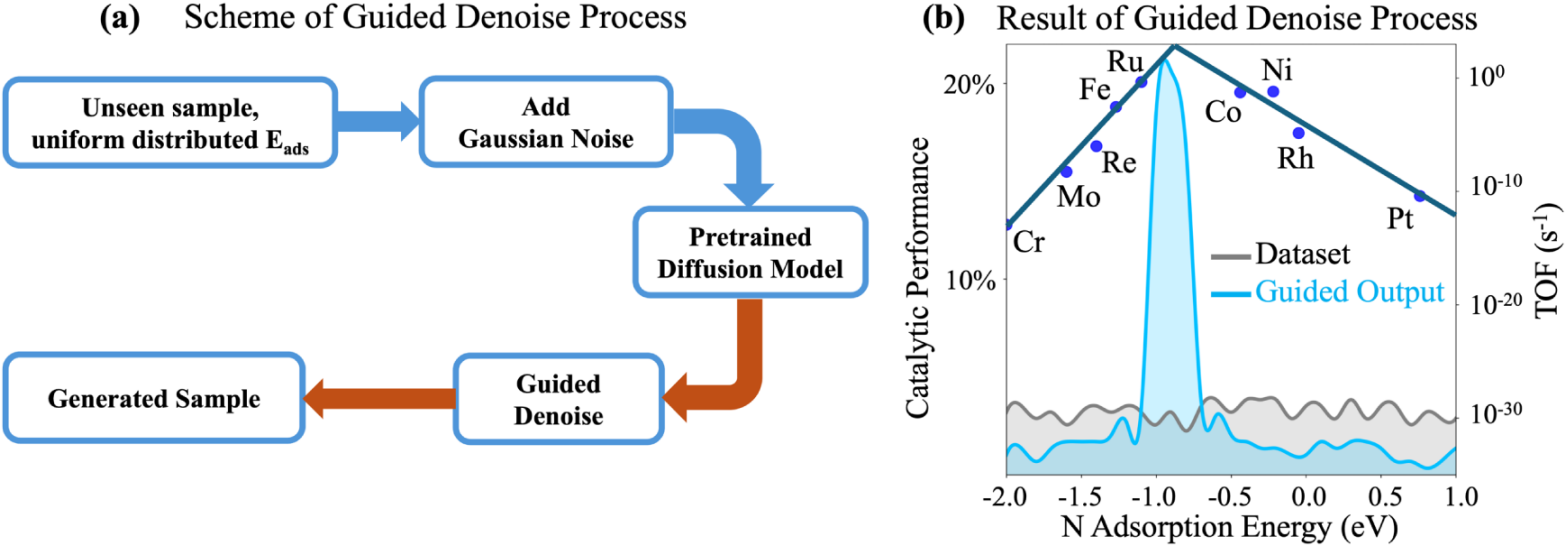
Data distribution after introducing ML-guided steps into the denoising process. (a) Schematic illustration of the ML-guided denoising workflow, where uniformly distributed unseen samples are used as initial inputs, and generated samples are near the targeted catalytic activity, represented by the targeted N adsorption energies. (b) Distribution of generated samples from the ML-guided denoising process. Gray histogram: *E*_N_ distribution of the initial input samples. Blue histogram: *E*_N_ distribution after guided denoising.

### DFT and Experimental Validation

Using the proposed inverse design workflow, we identified 13 bimetallic alloy candidates with outstanding predicted catalytic performance for ammonia decomposition. In this study, we focus on earth-abundant bimetallic alloys to ensure environmental sustainability and cost-effectiveness. Among the generated candidates from the diffusion model, compositions such as Co_34_Fe_66_, Ni_22_Fe_78_, Co_84_Mo_16_, Ni_83_Mo_17_, and Fe_67_Cu_33_, along with 8 additional alloys featuring diverse elemental ratios and adsorption sites, exhibited nitrogen adsorption energies near the optimal value of −0.9 eV (Table 2).

**Table 2:** Inverse Design Screened Bimetallic Alloy Catalysts Compared with DFT Validation

Bimetallic Alloy Catalysts	*E*_N_ML_ (eV)	*E*_N_DFT_ (eV)
Fe_78_Ni_22_	–0.82	–0.72
Fe_66_Co_34_	–0.97	–1.02
Fe_67_Cu_33_	–1.01	–1.04
Co_84_Mo_16_	–0.78	–0.8
Fe_56_Co_44_	–0.83	–0.74
Ni_83_Mo_17_	–0.87	–0.93
Co_75_Mo_25_	–0.93	–0.92
Cu_72_Mo_28_	–0.93	–0.95
Cu_48_Mo_52_	–0.94	–0.92
Cu_58_Cr_42_	–0.89	–0.94
Co_71_Cr_29_	–0.93	–0.89
Ni_84_Cr_16_	–0.92	–0.94
Co_89_Cr_11_	–0.81	–0.86

To validate these results, we first conducted DFT calculations for all 13 candidates, evaluating the nitrogen adsorption energetics at all relevant catalytic sites. For each alloy, we first identified the database model whose A:B ratio most closely matches the target compositions of these 13 candidates. Then, we generated 20 surface configurations for each target composition by randomly substituting A atoms with B in the database models (Figure S1). A comparison between DFT-calculated and ML-predicted adsorption energies is shown in Figure 4a and Table 2, revealing strong agreement and confirming the reliability of the inverse design process.

**Figure 4:**
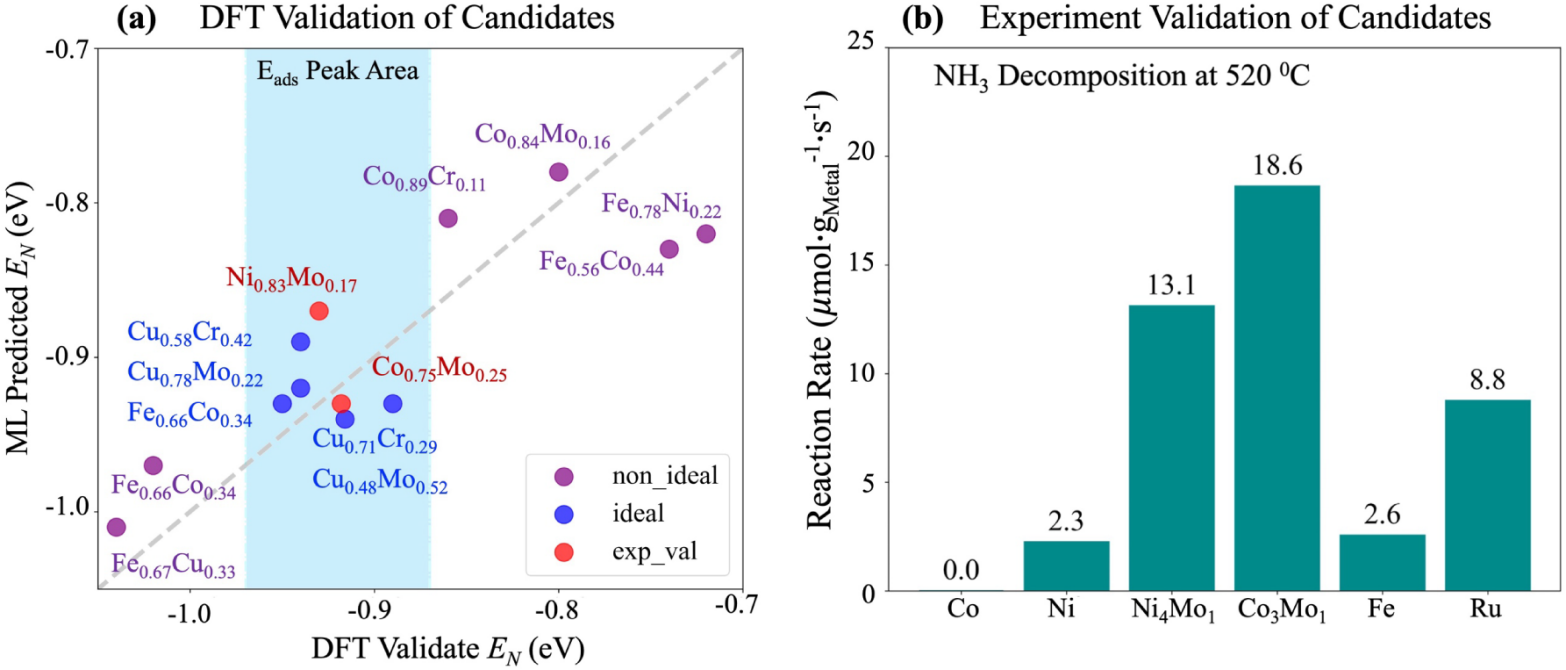
DFT and experimental validation of inverse design-guided bimetallic alloy catalysts. (a) DFT-calculated nitrogen adsorption energies for promising candidates generated by the inverse design framework. The blue-shaded region represents the ideal adsorption energy range (“*E*_ads_ Peak Area”). Blue dots indicate the most promising candidates, purple dots represent those with suboptimal performance, and red circles highlight the top candidates that were further validated experimentally. (b) Experimental evaluation of reaction rates for the promising candidates identified by inverse design. Reaction conditions: 500 ppm of NH_3_, weight hourly space velocity (WHSV) = 400,000 mL·g^–1^·h^–1^, reaction temperature = 520 °C.

Because surface segregation can be frequently observed in bimetallic catalysts and can markedly alter local surface composition and site identity,^37^ we further examined the segregation of the NiMo alloy, which is one of the most active candidates from our AI model. Our results show that the uniquely mixed AB alloy slab models are more stable than the segregated configurations by 0.45, 0.53, and 0.50 eV per Mo atom for Ni_15_Mo, Ni_7_Mo, and Ni_3_Mo, respectively, demonstrating that segregation of Mo is not preferred in the NiMo alloy system (Figure S8).

To experimentally verify the AI-designed catalysts, we synthesized and tested selected alloys, focusing on the NiMo system as a representative case. The influence of the Ni/Mo molar ratio on NH_3_ decomposition activity was systematically examined. As shown in Figure S10, the catalytic activity increased substantially with decreasing Ni/Mo ratio, demonstrating the synergistic advantages of Ni–Mo alloy formation and identifying the optimal composition.

Subsequently, we prepared and tested Ni_4_Mo_1_ and Co_3_Mo_1_ bimetallic catalysts alongside their monometallic Ni and Co counterparts. The inverse-designed alloys exhibited remarkable performance enhancement, as evidenced in Figures S11 and 4b, with reaction rates 5–7 times higher than that of the pure Ni or pure Co catalysts. DFT calculations attributed this improvement to synergistic alloying effects, showing nitrogen adsorption energies near the optimal value of −0.9 eV at the alloy sites, thereby validating the predictive capability of our diffusion model. Notably, although the activity trends were temperature-dependent, the alloy catalysts outperformed the Fe and Ru reference catalysts at the investigated temperature (Figure 4b).

Among these, the Co_3_Mo_1_ catalyst achieved a reaction rate of 18.6 μmol g_metal_^–1^ s^–1^, twice that of pure Ru at 520 °C, demonstrating the potential of these bimetallic systems as cost-effective alternatives to noble metal catalysts for high-temperature NH_3_ decomposition. XRD characterization (Figure S12) confirmed the bimetallic alloy structure of the Co_3_Mo_1_ catalyst, as the diffraction peaks were distinctly different from those of the individual metals. It should be noted that the samples were found in oxidized phases even after reduction treatment, which occurred due to the exposure to air during transfer before XRD measurement. The consistency between machine learning predictions, DFT simulations, and experimental outcomes highlights the effectiveness of the generative diffusion model for catalyst discovery.

### Mechanistic Insights into NiMo Catalytic Activity

To gain chemical insight into why the Ni_4_Mo system, as predicted by the AI inverse design and subsequently validated through DFT calculations and experimental measurements, exhibits high catalytic activity toward NH_3_ decomposition, we performed additional DFT simulations. The slab model was constructed by evaluating 60 configurations generated via random substitution of Mo with Ni atoms on the Ni_4_Mo­(111) surface (Figure S13a). In the most stable Ni_4_Mo­(111) configuration, two Mo atoms occupy the top layer (Figure 5).

**Figure 5:**
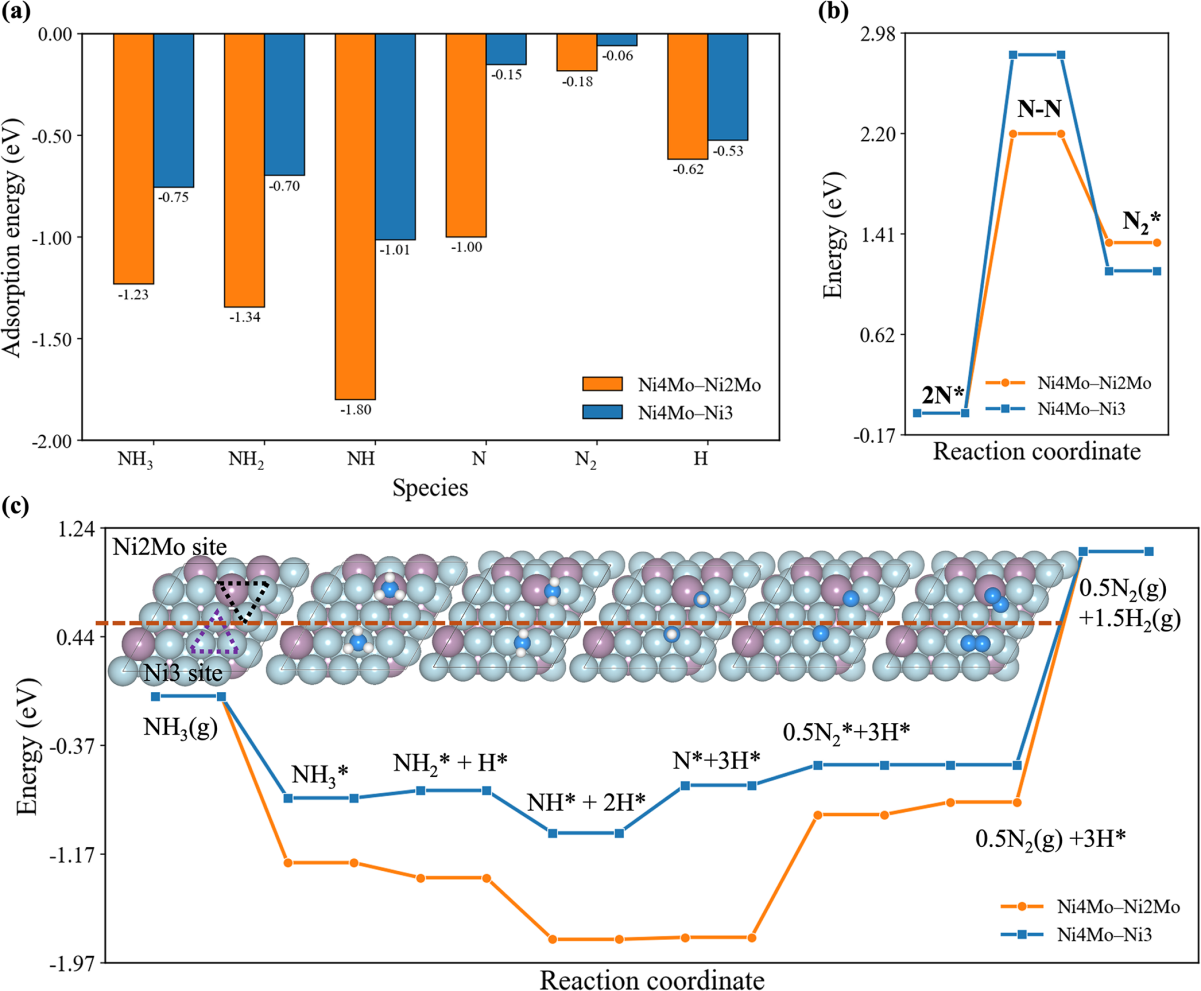
Comparison of adsorption geometries (a), reaction profile (b), and N–N coupling (c) at the Ni_2_Mo and Ni_3_ sites on Ni_4_Mo­(111). Color code: Ni, light blue; Mo, light purple; N, blue; H, white.

NH_3_ decomposition was then examined at two representative hollow sites, Ni–Mo–Ni (Ni_2_Mo) and Ni_3_. The Ni_3_ site can represent the performance of pure Ni catalysts. Bader charge analysis (Figure S13b) indicates that for the Ni_2_Mo site, Mo and Ni atoms carry charges of +0.51 |e| and −0.15 |e|, respectively, suggesting stronger NH_3_ adsorption on Mo due to electrostatic attraction between the positively charged Mo site and the negatively polarized N atom of NH_3_. The *d*-band centers are shown (Figure S13c) for Mo (−0.56 eV) and Ni (−0.80 eV), both referenced to a Fermi level of 0 eV, further confirming stronger adsorbate-metal interactions at the Mo site, consistent with the DFT-calculated adsorption energies of NH_3_ decomposition intermediates (Figure 5a and Table S1).

From a kinetic perspective, N≡N bond formation, the rate-limiting step identified in our previous work,^21^ is kinetically more favorable on the Ni_2_Mo site, with an activation barrier of 2.2 eV compared to 2.8 eV on Ni_3_ (Figure 5b). Moreover, we further analyzed the reaction network from a thermodynamic perspective by comparing all elementary steps on the Ni_3_ and Ni_2_Mo hollow sites of the Ni_4_Mo­(111) surface. NH_3_ decomposition over the Ni_2_Mo site is more exothermic than that on Ni_3_, reflecting a more favorable thermodynamic driving force (Figure 5c). These results demonstrate that Mo incorporation into the Ni surface markedly enhances the catalytic performance for NH_3_ decomposition by (i) facilitating NH_3_ adsorption and stabilizing key intermediates and (ii) lowering the activation barrier for N≡N bond formation rate-limiting step.

## Conclusion

In summary, we have presented a machine learning-guided diffusion framework for the inverse design of bimetallic alloy catalysts, with a specific focus on optimizing nitrogen adsorption energy for ammonia decomposition. By integrating generative modeling with predictive ML evaluation, our approach efficiently navigates the vast compositional and structural space of bimetallic systems, identifying candidates with near-optimal catalytic performance.

The strong agreement among ML predictions, DFT simulations, and experimental validations underscores the robustness and practical utility of the proposed workflow. Beyond the specific case of ammonia decomposition, this framework is potentially broadly applicable to other catalytic reactions and complex material systems, including high-entropy alloys and multicomponent surfaces.

As a future enhancement to the inverse design workflow, we plan to incorporate a standardized classifier-free guidance mechanism as the property guidance function. Classifier-free guidance provides a versatile and robust approach for steering diffusion models, enabling the generation of complex materials that satisfy specific requirements and constraints without relying on external classifiers.^29^ Building on this foundation, the machine-learning-guided diffusion framework can be extended to address more challenging design problems, such as the discovery of high-entropy alloys optimized for ammonia decomposition. By combining scalability, accuracy, and chemical relevance, this methodology offers a powerful and generalizable tool for accelerating catalyst discovery and advancing data-driven materials design in catalysis.

## Methods

### Computational Setups

DFT calculations were performed using the Vienna Ab-initio Simulation Package (VASP) to evaluate the energetics (adsorption, reaction energetics, and activation barriers) of NH_3_ decomposition over different catalysts. The calculations utilized Perdew–Burke–Ernzerhof (PBE) functionals and the projector-augmented wave (PAW) methods to model electron exchange-correlation and ion-electron interactions.^38–40^ The bulk and surface models of the pure metals (Cr, Fe, Co, Ni, Cu, Mo, Ru, Rh, Pd, Ag, W, Re, Os, Ir, Pt, and Au) and their bimetallic alloys (835 in total) with ratios of (1:1, 3:1, 7:1, and 15:1) were constructed via the Atomic Simulation Environment (ASE)^41^ and subsequently optimized. A Monkhorst–Pack mesh with (3 × 3 × 1) *k*-points and a plane-wave energy cutoff of 450 eV were applied to the four-layer *p*-(4 × 4) metallic surfaces. The top two layers of these metallic surfaces (835 in total) were allowed to relax to facilitate their electronic interactions with the adsorbates, while the bottom two layers were fixed at their bulk positions. For screening the N adsorptions, we focus on the hollow sites, and the configurations (∼3800) were optimized with the energy and force criteria of 10^–5^ eV and 0.03 eV/Å,^42^ respectively. Spin polarization was considered, and the density of states related properties were obtained by Pymagen.^43^ More details regarding the DFT calculations on N adsorption over both monometallic and bimetallic alloys are given in Supporting Information Section S1.

## Materials

Commercial SiO_2_ support (surface area = 180 m^2^/g) was obtained from Evonik. Cobalt­(II) nitrate hexahydrate (>99%), nickel­(II) nitrate hexahydrate (>99%), ammonium molybdate­(VI) tetrahydrate (>99%), iron­(III) nitrate nonahydrate (>99%), and ruthenium­(III) nitrosylnitrate were purchased from Fisher Scientific. All chemicals were used without further treatment.

### Catalyst Synthesis

The metal and alloy catalysts were prepared using the incipient wetness impregnation (IWI) or co-impregnation (co-IWI) method. Basically, an aqueous solution of cobalt nitrate, nickel nitrate, or their mixture with ammonium molybdate was added dropwise to SiO_2_ under stirring. The total metal loading was fixed at 10 wt %, with molar ratios of Ni/Mo = 15/1, 7/1, or 4/1, and Co/Mo = 3/1 for the alloy catalysts. After impregnation, the materials were dried at 120 °C for 1 h and then calcined in air at 550 °C for 2 h with a heating rate of 5 °C/min. For comparison, an Fe catalyst on SiO_2_ with 10 wt % Fe using iron­(III) nitrate nonahydrate precursor and a Ru catalyst on SiO_2_ with 2 wt % Ru using ruthenium nitrosylnitrate precursor were prepared using the same process as for single metal catalysts. The resulting catalysts are denoted as Co, Ni, Ni_15_Mo_1_, Ni_7_Mo_1_, Ni_4_Mo_1_, Co_3_Mo_1_, Fe, and Ru, respectively.

### Catalytic Testing

The catalytic activity evaluation for the NH_3_ decomposition over all catalysts was conducted by using a continuous flow fixed-bed quartz tubular microreactor with an internal diameter of 4.0 mm. For each test, 30 mg of catalyst (40–60 mesh) was diluted with 0.25 g of inert SiC (40–60 mesh) to minimize the effect of hot spots. Prior to testing, the catalyst was reduced in a 10% H_2_/Ar flow at 500 °C for 2 h to form the active metal or alloy phase. The reaction gas comprised 500 ppm of NH_3_ using Ar as the balance. The total flow rate was controlled at 200 mL/min, resulting in a weight hourly space velocity (WHSV) of 400,000 mL·g^–1^·h^–1^. Temperature-programmed testing employed a heating rate of 2 °C/min. Reactants and products were analyzed online by a Nicolet iS20 FTIR spectrometer with a gas cell (200 mL volume) of 2 m path length. The reactant conversion was defined as (*c*_inlet_ – *c*_outlet_)/*c*_inlet_ × 100%, where *c*_inlet_ and *c*_outlet_ were the inlet and outlet NH_3_ concentration in the feed stream, respectively.

## Supplementary Material





## Data Availability

The data used for developing machine learning models and experimental work are available in the Supplementary Data file.

## Notes

Machine learning algorithms are included as python modules and data files used to create all the plots (ZIP)
Additional computational details, machine learning model architectures, feature importance analysis, and additional experimental details, including activity measurements and XRD characterization (PDF)
