# Supplementary material for: Diffusion Model-Guided Inverse Design of Bimetallic Catalysts for Ammonia Decomposition
Source: J Am Chem Soc. 2025 Dec 19;148(1):537–46. doi: 10.1021/jacs.5c14652 (PMC12814173; doi:10.1021/jacs.5c14652)
Supplement: Supplementary file 2 [file ja5c14652_si_002.pdf]

Diffusion Model-Guided Inverse Design of Bimetallic Catalyst for Ammonia Decomposition

Jiaqi Yang[1], Kailong Ye[3], Shaohua Xie[3], Qiang Li[2], Charles Milhans[2], Fudong Liu[3]*, Fanglin Che[1,2]*

[1]Department of Chemical Engineering, Worcester Polytechnic Institute, Worcester, MA 01609, USA.
[2]Department of Chemical Engineering, University of Massachusetts Lowell, Lowell, MA 01854, USA.
[3]Department of Chemical and Environmental Engineering, Bourns College of Engineering, Center for Environmental Research and Technology (CE-CERT), Materials Science and Engineering (MSE) Program, UCR Center for Catalysis, University of California, Riverside, Riverside, CA 92521, USA.
*Corresponding Authors: fudong.liu@ucr.edu; fche@wpi.edu

1. Computational Details

DFT Method: The density functional theory (DFT) calculations were performed using the Vienna *Ab*-initio Simulation Package (VASP)[1-3]. DFT calculations were used to calculate the adsorption energies for binding energy of nitrogen. The adsorption energy[4] (E_{ads}) was calculated using **Equation S1**:

$$E_{ads} = E_{total} - E_{surface} - E_{adsorbate} \quad \text{(S1)}$$

here E_{total} corresponds to the total energy of the adsorbed species on the surface, $E_{surface}$ represents the energy of the clean surface without the adsorbate, and $E_{adsorbate}$ denotes the energy of the adsorbate in the gas phase. For example, to calculate the binding energy of N*, $E_{adsorbate}$ refers to the energy of half of the N_2 molecule in the gas phase.

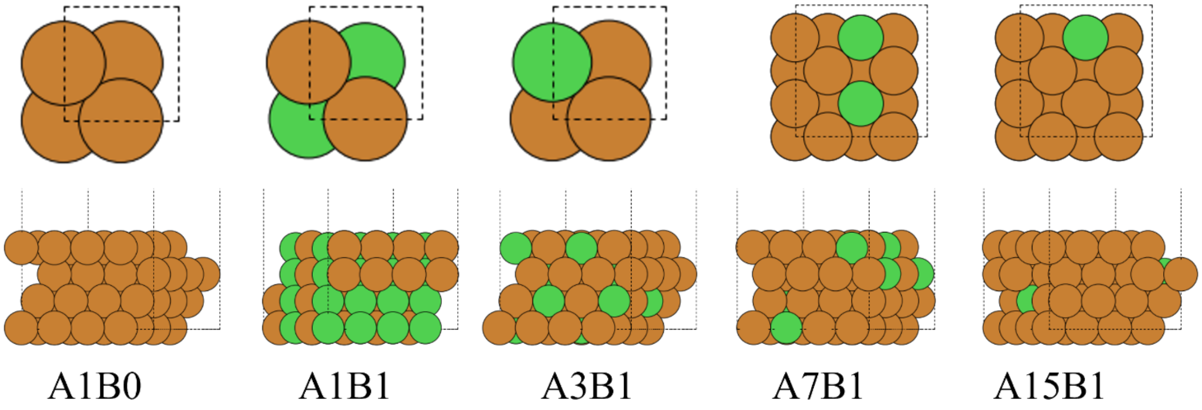

Figure S1. Bulk and slab models of pure metal and bi-metallic alloys.

DFT Models: For consistency, all slab models constructed in this work are based on face-centered cubic (FCC) crystal structures, which serve as the structural foundation for both pure metals and

bimetallic alloys. For the pure metal systems, the modeling process began with conventional unit cells containing four metal atoms. These bulk structures were first fully optimized to obtain the equilibrium lattice constants. Subsequently, the low-index (111) surfaces were generated by cleaving the relaxed bulk structures along the (111) crystallographic plane, using the Atomic Simulation Environment (ASE) package. To construct bimetallic alloy models, a substitutional approach was adopted. The host atoms in the pure metal bulk were systematically replaced by secondary metal atoms to achieve the desired alloy compositions. Starting from the 4-atom conventional cell, substitution yielded alloy bulk structures with 1:1 and 3:1 metal ratio. To access lower concentrations such as 7:1 and 15:1, the 4-atom bulk structures were periodically extended—doubled along the x and y direction to form 16-atom cells, with two and one host atom being replaced to achieve the 7:1 and 15:1 ratio and then expanded in z directions to make a 32-atom bulk structure. All constructed bulk structures, including alloy supercells, were fully relaxed to determine their equilibrium configurations.

All (111) slab models consisted of four atomic layers, each containing 16 atoms, similar to a p(4×4) periodicity on the (111) surface of the pure metal. It should be noted that the alloy composition is uniformly distributed across all layers, preserving the specified atomic ratio throughout the slab thickness. This setup ensures structural consistency and sufficient lateral dimensions to minimize interaction between periodic images during adsorption and surface reaction modeling. All slab models were subsequently optimized. During these optimizations, the bottom two atomic layers were fixed, while the upper two layers were allowed to relax. For the N* adsorption studies, we systematically investigated various hollow sites, which are labeled using the convention: "composition_site_bottom atom". For example, in the A15B1 alloy system, the considered hollow sites include: A3_fcc_A, A3_hcp_A, A3_hcp_B, Ag2B_fcc_A, Ag2B_hcp_A. In this labeling scheme: the prefix (e.g., A3 or Ag2B) denotes the three surface atoms forming the hollow site and their elemental identity. The middle term (fcc or hcp) indicates the type of hollow site geometry. The suffix (e.g., _A or _B) identifies the elemental type of the third-layer atom directly beneath the hollow site. Another example is the A3B1 surface shown in the **Figure S2**, there are two fcc hollows and two hcp hollows, distinguished by the composition of the surface triangle (A3 vs A2B1) and by the identity of the atom directly beneath the hollow (third layer for fcc, second layer for hcp). Specifically, the two fcc sites are A3_fcc_B) and A2B1_fcc_A, and the two hcp sites are A3_hcp_B) and A2B1_hcp_A), where the notation indicates the surface-triangle composition, type, and the underlying atom. In our database, each N adsorption entry is labeled by this triplet: surface-triangle composition (A3 or A2B1), site type (fcc or hcp), and underlying atom (A or B). And the full set of labeled configurations was used to train our model. Notably, this triplet also serves as the prediction target of our diffusion model; that is, the model jointly predicts the alloy composition and the full site descriptor (site type, surface-triangle composition, and underlying atom).

Regarding the segregation, it is commonly observed in bimetallic alloys, however, it is also another scenario of the structure complexity of the catalyst structures.[5] We did not consider the segregation in present work due to the large chemical space when introducing the segregation with/without species-induced to the database for our AI model. Another important reason for not considering the separation is the fact that we used N adsorption energy over the surface hollow sites as the key feature to guide our AI model and the hollow sites of segregated surfaces will be represented by the alloy systems with various ratios. For instance, once the segregation occurs for metal A in the

AB alloy, the surface hollow sites will be composed by three A atoms, which is similar to the A-rich alloy systems like A15B1, and A7B1.

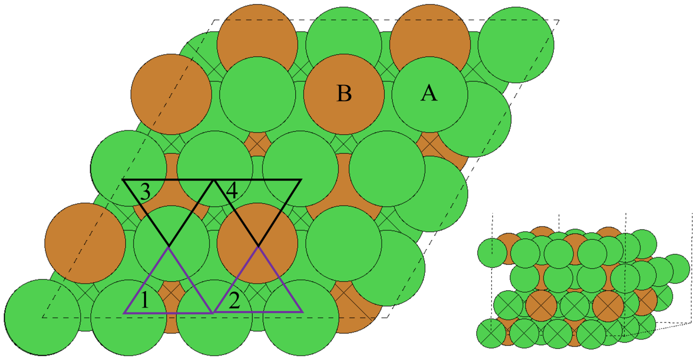

	Site type	Site composition	Underlying atom
1	fcc	A3	B
2	fcc	A2B1	A
3	hcp	A3	B
4	hcp	A2B1	A

Figure S2. Surface hollow sites of the A3B1(111) surface. The atoms A and B of A3B1(111) model are in green and brown.

It should be noted that step/edge ensembles (*e.g.*, the B5 site on Ru) can be more active for the 2N* association than close-packed (111) terraces, owing to their geometry and the presence of low-coordinated atoms. However, our objective in present is to predict activity trends across a broad set of bimetallic alloys under a controlled, internally consistent setting; accordingly, we restrict the surface termination to flat fcc(111) and evaluate N adsorption in the low-coverage limit at all symmetry-inequivalent threefold hollows (fcc/hcp). This choice is supported by the persistence of volcano-type trends for NH_3 decomposition across stepped (211) facets.[3] To further confirm this trend, we performed additional calculations microkinetic simulation for step surface sites across different catalysts, using the DFT data from Wang et al.[6] The results show a consistent volcano-type correlation between activity and EN, again with an optimal value near Ru (approximately -0.90 eV over both terrace and step sites, as shown in **Figure S3**), confirming that nitrogen adsorption energy remains the key descriptor governing ammonia decomposition activity on both terrace and step surfaces. This consistency indicates that relative activity trends are largely independent of catalyst morphology. In our collaboration with experiments,[3] we observed that DFT and experimental volcano plots agree on activity trends when using (111) slabs, indicating that terraces suffice for ranking catalysts, although step sites may still influence absolute rates. Therefore, in this work, we focused on terrace sites as a representative and computationally feasible model, evaluating E_N across all symmetry-inequivalent threefold hollow sites (fcc and hcp) to ensure a consistent and physically meaningful comparison among different bimetallic alloys. To

avoid a combinatorial explosion from steps, defects, and facet distributions in the database construction and to enable a tractable database for model training, we therefore compare alloys on (111) terraces only. We view this study as a prototype for integrating AI into catalytic screening; extension to stepped/defective surfaces will be pursued in future work by expanding the database.

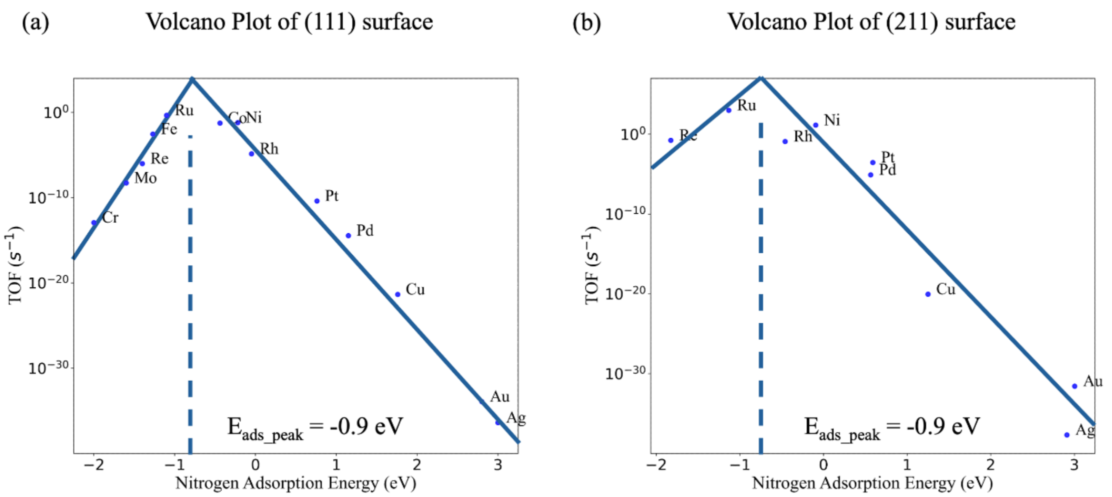

Figure S3. A volcano-shape relationship between the catalytic activity (TOF) of NH_3 decomposition and the adsorption energy of nitrogen over (a) terrace[3] and (b) step surfaces across various metal catalysts. The temperature for performing the MKM is at 520 °C. The DFT data for the step sites of different catalysts were extracted from Wang *et al.*[6].

For the coverage impacts, despite the fact that N binding energy will become much weaker with increasing the surface coverages. To reduce the complexity from this layer, we have examined the N adsorption at various N coverages for the NiMo system. As shown in **Figure S4**, our calculations for the NiMo system at different nitrogen coverages confirm that the overall trend in E_N remains unchanged across surfaces, supporting the assumption that coverage effects do not alter the comparative activity ranking among catalysts. This evidence supports our goal to compare the activity trend of various bimetallic alloy systems using the N adsorption energy as the key descriptor.

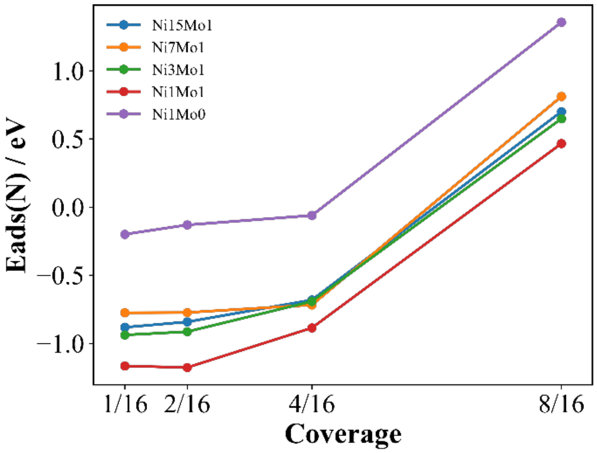

Figure S4. N adsorption energy on various NiMo alloy surfaces with coverage increasing from 1/16 to 8/16 monolayer.

2. Details of Machine Learning Descriptors

To transform the DFT simulated bimetallic structures to ML inputs, we designed a series of descriptors, which include multiple aspects of the bimetallic alloy samples. The descriptors are composed of four major parts: (1) elemental composition, (2) atomic properties, (3) *d*-band filling, (4) adsorption site characteristics. The detailed features included in the descriptors are in **Table 1**. These four aspects generally cover all structure details related to N* adsorption during NH_3 decomposition, which uniquely defined each bimetallic system during the reaction.

3. Volcano Plot of Ammonia Decomposition Reaction

In our previous work[3], we utilized DFT simulations and MKM simulations to calculate a volcano relationship to link catalytical performance of ammonia decomposition with N* adsorption energy. The catalytical performance is evaluated by the turnover frequency (TOF). TOF is derived by dividing the number of NH_3 molecules converted per unit time by the number of active sites.

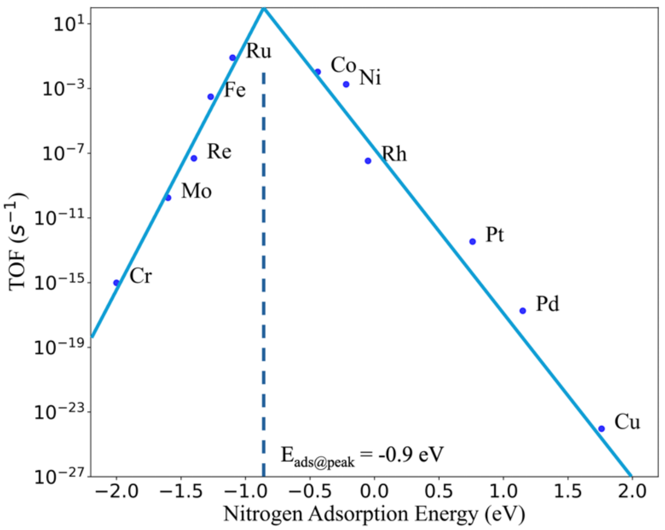

Figure S5. Multi-scale model derived volcano plot of ammonia decomposition at 520 ºC.

4. Feature Importance Analysis for Predictive Model

SHapley Additive exPlanations (SHAP)[7] has been widely applied to identify important features for machine learning (ML) models. Hence, we applied SHAP analysis on our forward predictive model for N adsorption energy. The SHAP analysis identified the following features as the most important features: *d*-band filling of 1st nearest neighbor (f_a, f_b, f_c, f_d), spatial extent (SE), electron affinity (EA), ionization energy (IE).

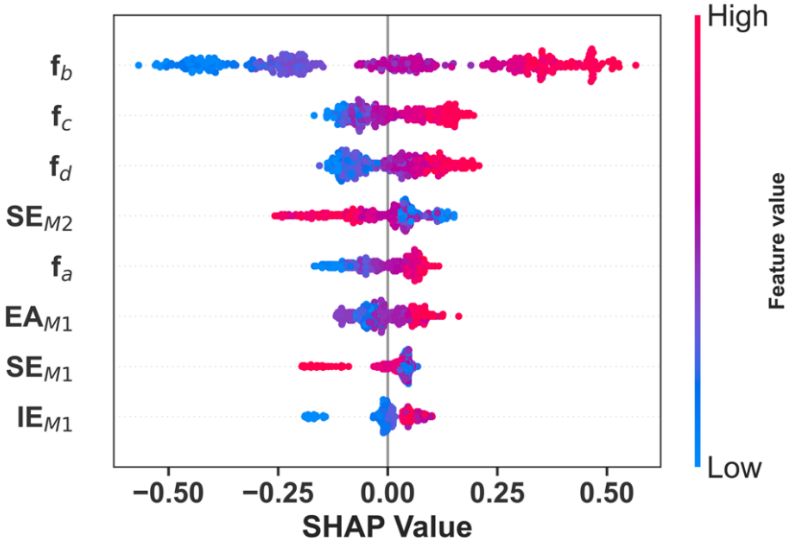

Figure S6. Interpretable explanation of feature importance through SHAP Beeswarm Plot.

5. Modifications of Loss Function

The diffusion model is trained to learn the noise patterns between these noised matrices by minimizing the discrepancy between the predicted and actual noise added during the perturbation

process. The detailed process is following the model introduced by Ho et al.[8] The training objective is formalized through the loss function shown in **Equation S2**,[8]

$$L_{simple,t} = E_{x0\sim q;z\sim N(0,1)}[||\epsilon_\theta(x_t,t) - z||^2] \quad \text{(S2)}$$

Where $\epsilon_\theta(x_t,t)$ is the model output noise vector and z is the trackable gaussian noise. $E_{x0\sim q;z\sim N(0,1)}$ is the expectation over integrals of term $||\epsilon_\theta(x_t,t) - z||^2$.

To introduce physics restraints of all bimetallic system, we extend the loss function with an extra term to reject the results violating restraints, shown in **Equation S3**:

$$L_{restraints,t} = L_{simple,t} \times ReLU(f_{out}) \quad \text{(S3)}$$

Here, we use a rectified linear unit (ReLU) function to add extra loss for results violating restraints. The term 'f_{out}' is showing the amount of feature f (belongs to range [a, b]) deviate from the designated range, shown in **Equation S4**:

$$f_{out}\begin{cases} |f-b|, & f > b \\ 0, & f \in [a,b] \\ |f-a|, & f < a \end{cases} \quad \text{(S4)}$$

To introduce the guidance of ML predictive model, we introduce a guidance term on the loss function, shown in **Equation S5**:

$$L_{guidance,t} = L_{simple,t} \times |E_{ads_{ML}} - E_{ads_{IDEAL}}| \quad \text{(S5)}$$

About the prediction of E_{adsML} for the structure, it is evaluated by the ML forward model at every denoised step t. In this approach, the E_{adsML} will guide the denoise via augmenting the Loss function. The closer between the E_{adsML} and ideal value (i.e., the binding energy of nitrogen of -0.9 eV), a bigger reward will be added to the loss. This is a plain way to generate a sample with desired properties.

6. Reconstruction Generates Multiple Atomic Configurations with Identical Local Adsorption Sites.

(1) DFT simulations on different configurations with fixed composition and local sites

For alloy catalysts in our work, we assume that the local adsorption-site environment, particularly the identity and arrangement of first-nearest neighbors, plays a dominant role in determining the binding energies of reaction intermediates, such as nitrogen.

To validate this assumption, we performed DFT simulations on a representative Co_3Mo slab, one of the most active candidates generated by our diffusion model. Starting from a non-segregated

Co_3Mo surface, we examined a three-fold hollow (hcp) adsorption site composed of two Co atoms and one Mo atom as first-nearest neighbors. While keeping both the overall composition and the first nearest-neighbor atoms fixed, we randomly permuted the remaining atoms to create 20 distinct configurations.

DFT calculations of nitrogen adsorption energies for these configurations (**Figure S7**) yielded values ranging from -0.78 eV to -1.22 eV, with a standard deviation of 0.11 eV, comparable to typical DFT uncertainties. This narrow variation confirms that catalytic performance remains largely invariant when the first-nearest neighbors and composition are fixed. Because our descriptor matrix uniquely defines the local adsorption site by its first nearest neighbors and local site geometry, these results validate that the reconstruction procedure generates multiple atomic configurations sharing equivalent adsorption environments and thus physically meaningful catalytic behavior.

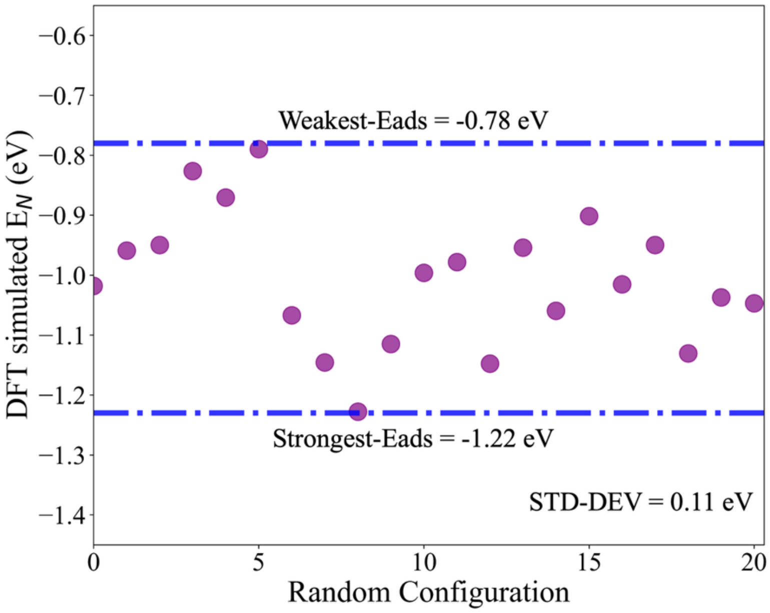

Figure S7. N adsorption energy distribution plots for 20 random configurations, with the weakest E_N as -0.78 eV, the strongest E_N as -1.22 eV and a standard deviation of 0.11eV, comparable to typical DFT uncertainties.

(2) DFT simulations on surface segregation

Another constraint imposed in our reconstruction procedure is the assumption that the alloys exhibit minimal surface segregation (i.e., uniformly mixed models in **Figure S8**). We acknowledge that surface segregation is a common phenomenon in bimetallic alloys and represents an additional layer of structural complexity in catalytic systems. Such surface segregation can also be influenced by adsorbate-induced effects, particularly under reaction conditions.[5]

In the present work, we did not explicitly include surface segregation in our AI training framework for two main reasons. First, introducing segregation, with or without species-induced effects, would substantially expand the chemical and structural space, making it computationally prohibitive to construct a representative database for training. Second, our AI model employs the nitrogen adsorption energy on surface hollow sites (hcp) as a key guiding descriptor. In segregated surfaces, hollow sites dominated by one metal species (e.g., three identical atoms from a segregating element) are effectively represented in our database by alloy systems with higher concentrations of that element (e.g., $A_{15}B_1$ or A_7B_1 for an A-segregated surface in an AB alloy). Thus, the essential surface environments arising from segregation are already implicitly captured within our compositional sampling.

We conducted DFT simulations on the Ni-Mo alloy system, one of the most active candidates predicted by our AI model. As shown in **Figure S8**, the uniformly mixed NiMo slabs are energetically more stable than their segregated counterparts by 0.45, 0.53, and 0.50 eV per Mo atom for $Ni_{15}Mo$, Ni_7Mo, and Ni_3Mo, respectively. These results confirm that Mo segregation to the surface is thermodynamically unfavorable, supporting the validity of the minimal-segregation assumption adopted in this study.

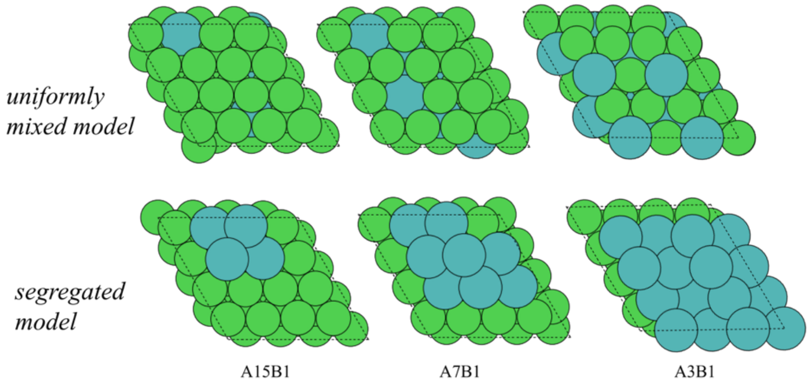

Figure S8. Uniformly mixed model (present work) and the segregated model. For NiMo alloys. The uniquely mixed models are more stable than the segregated configurations by 0.45, 0.53, and 0.50 eV per Mo atom for $Ni_{15}Mo$, Ni_7Mo, and Ni_3Mo, respectively. Color code: Green: Ni (metal A); Cyan: Mo (metal B).

Furthermore, we generated 40 random surface-segregated configurations for the Co_3Mo system, another highly active candidate predicted by our diffusion model. These configurations preserved the same local adsorption sites as those analysed in **Figure S8**, while allowing random Co or Mo segregation on the top two surface layers. As shown in **Figure S9**, the segregated structures are thermodynamically less stable and exhibit substantial variations in adsorption energies of nitrogen, indicating a notable deterioration in catalytic performance. Therefore, under the assumptions of

minimal-segregation constraint, surface segregation can be regarded as a secondary factor with only minor influence on the overall catalytic behavior.

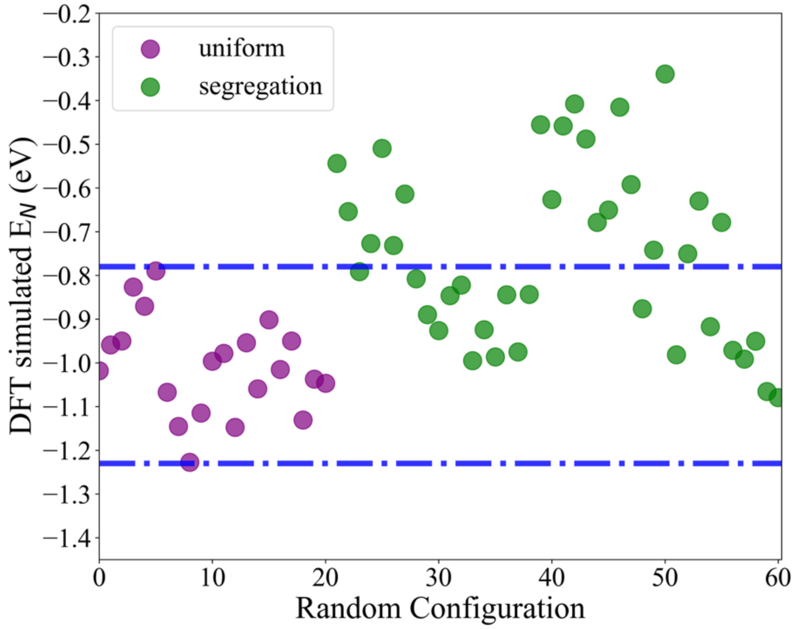

Figure S9. N adsorption energy distribution plots for 20 random uniform configurations (minimal-segregation constraint) and 40 random configurations with surface segregation. Purple: samples with minimal-segregation constraint. Green: samples with surface segregations.

(3) SHAP analysis on feature importance

The critical role of the first nearest-neighbor environment is further supported by the results of our forward ML model for predicting nitrogen adsorption energies. To interpret the model and quantify feature contributions, we employed SHapley Additive exPlanations (SHAP) analysis[7], a widely used method for assessing feature importance in ML models.

The SHAP results (**Figure S4**) identified the following as the most influential features: (1) d-band filling of the first-nearest neighbors (fa, fb, fc, fd); (2) spatial extent (SE); (3) electron affinity (EA); and (4) ionization energy (IE).

Among these, the d-band filling of the first-nearest neighbors, which is directly determined by the site type, identity of neighboring atoms, and overall composition, was found to be the dominant descriptor governing nitrogen adsorption behavior.

These findings confirm that our reconstruction procedure reliably produces DFT-ready structures with uniquely defined local adsorption site environments. The lack of strict one-to-one mapping between descriptor matrices and atomic configurations introduces only minor variations in catalytic performance under the assumptions of minimal surface segregation and fixed (111) facet.

We acknowledge, however, that a significant gap remains between DFT-simulated models and real-world experimental systems. Future developments will incorporate adsorbate coverage, structures (defects, segregations, and so on), and material synthetic feasibility into both simulation and ML workflows to more accurately reflect realistic catalytic environments. As an initial demonstration of diffusion model-based inverse design for alloy catalysis, our current reconstruction approach provides a robust and sufficient framework for bimetallic systems. In future work, we plan to extend this methodology using graph representations and graph neural networks (GNNs) to address more complex alloy systems, enabling greater structural fidelity and reduced uncertainty in configuration-dependent predictions.

7. Catalytic Performance of Bimetallic Alloy Catalysts

Using the NiMo catalyst system as a representative example, we investigated the influence of Ni/Mo molar ratio on NH_3 decomposition performance by synthesizing bimetallic alloy catalysts with Ni/Mo ratios of 15:1, 7:1, and 4:1. As demonstrated in **Figure S10**, the NH_3 decomposition activity markedly increased with decreasing Ni/Mo ratio, clearly highlighting the synergistic benefits of Ni-Mo alloy formation. Among these catalysts, the Ni_4Mo_1 catalyst was the optimal catalyst.

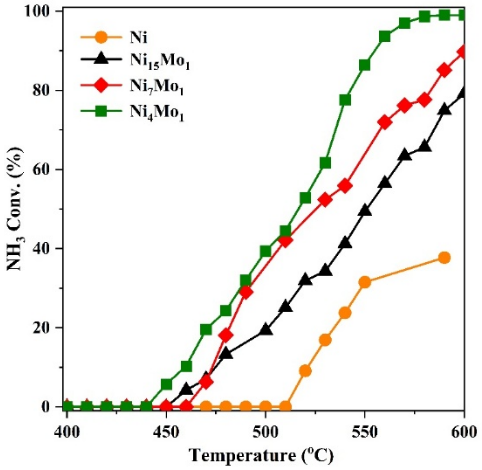

Figure S10. NH_3 decomposition activity on NiMo catalysts with Ni/Mo molar ratios of 15/1, 7/1, and 4/1. Reaction conditions: 500 ppm NH_3; weight hourly space velocity (WHSV) of 400,000 $mL\cdot g^{-1}\cdot h^{-1}$.

To validate promising bimetallic alloy candidates identified through Inverse Design, we prepared and evaluated Ni_4Mo_1 and Co_3Mo_1 catalysts along with their single-metal counterparts for NH_3 decomposition. As shown in **Figure S11**, the bimetallic alloy catalysts demonstrated significantly enhanced activity compared to pure Ni and Co catalysts. This improvement was attributed to the synergistic effects of metal alloying, as DFT calculations revealed that the alloy sites exhibited nitrogen adsorption energies near the optimal value of -0.9 eV. Among these catalysts, Co_3Mo_1 exhibited the best performance.

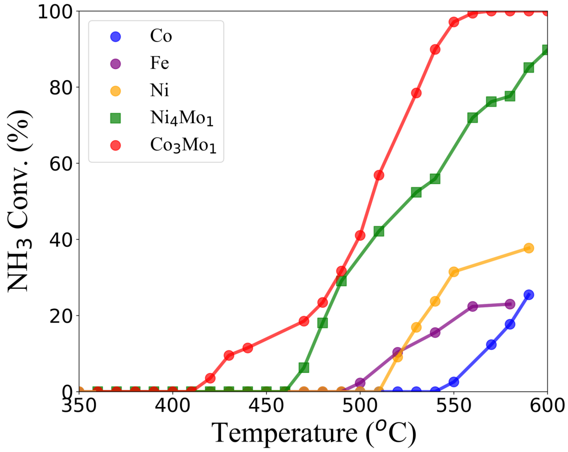

Figure S11. NH_3 decomposition activity on representative Ni_4Mo_1 and Co_3Mo_1 bimetallic alloy and single metal catalysts. Reaction conditions: 500 ppm NH_3; weight hourly space velocity (WHSV) of 400,000 $mL \cdot g^{-1} \cdot h^{-1}$.

To confirm the quality of the synthesized bimetallic alloys, XRD was performed on a typical CoMo sample and its single-metal counterparts. The results are presented in **Figure S12**.

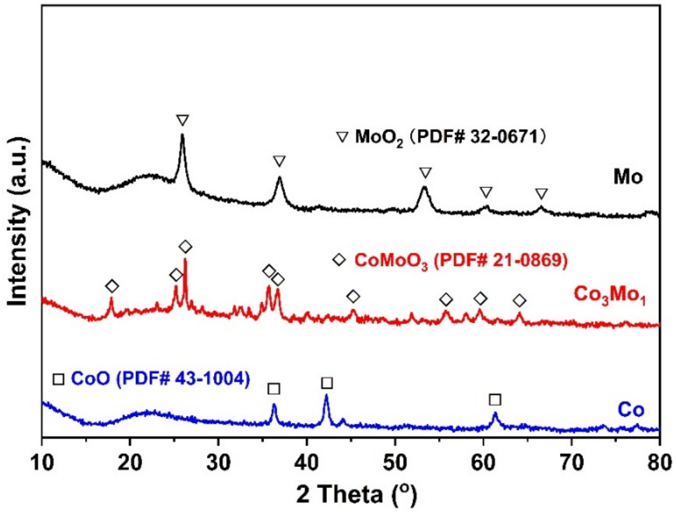

Figure S12. XRD patterns of Co, Co_3Mo_1, and Mo samples. Prior to *ex situ* XRD testing, the samples were reduced in 10% H_2/Ar flow at 500 °C for 2 h.

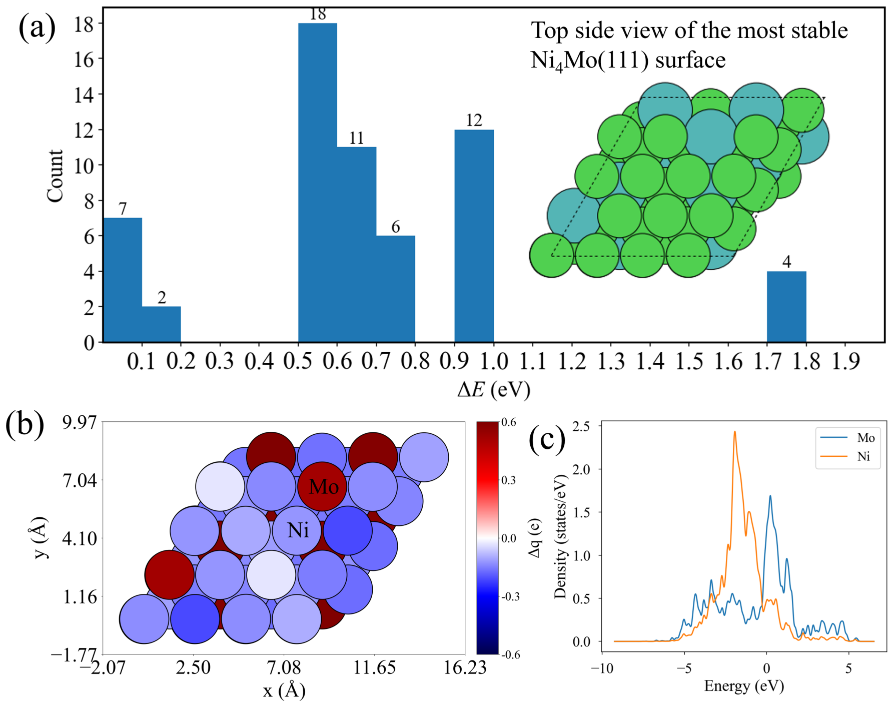

Figure S13. (a) Distribution of the relative energies of $Ni_4Mo(111)$ candidate structures with respect to the lowest-energy configuration. Color codes: Green: Ni; Cyan: Mo (b) Bader charge distribution of Mo and Ni atoms in the $Ni_4Mo(111)$ model. (c) Projected density of states of Mo and Ni atoms labeled in panel (b).

Table S1. Adsorption Energies of intermediates and dehydrogenation energies over the Ni_2Mo and Ni_3 sites of $Ni_4Mo(111)$ surface.

**Adsorption Energy (eV)**		
**Species**	**Ni_2Mo site**	**Ni_3 site**
NH_3	-1.23	-0.75
NH_2	-1.34	-0.70
NH	-1.80	-1.01
N	-1.00	-0.15
N2	-0.18	-0.06
H	-0.62	-0.53
**Reaction Energy (eV)**		
**Reactions**	**Ni_2Mo site**	**Ni_3 site**
NH_3* -> NH_2* + H*	-0.11	0.06
NH_2*->NH*+H*	-0.46	-0.31
NH*->N*+H*	0.01	0.35

References

(1) Che, F.; Gray, J. T.; Ha, S.; Kruse, N.; Scott, S. L.; McEwen, J.-S. Elucidating the roles of electric fields in catalysis: a perspective. *ACS Catalysis* **2018**, *8* (6), 5153-5174.
(2) Che, F.; Zhang, R.; Hensley, A. J.; Ha, S.; McEwen, J.-S. Density functional theory studies of methyl dissociation on a Ni (111) surface in the presence of an external electric field. *Physical Chemistry Chemical Physics* **2014**, *16* (6), 2399-2410.
(3) Ahmat Ibrahim, S.; Meng, S.; Milhans, C.; Barecka, M. H.; Liu, Y.; Li, Q.; Yang, J.; Sha, Y.; Yi, Y.; Che, F. Interpretable machine learning-guided plasma catalysis for hydrogen production. *Nature Chemical Engineering* **2025**, *2* (11), 699-710.
(4) Wan, M.; Yue, H.; Notarangelo, J.; Liu, H.; Che, F. Deep learning-assisted investigation of electric field–dipole effects on catalytic ammonia synthesis. *JACS Au* **2022**, *2* (6), 1338-1349.
(5) Wu, H.; Liu, C.; Guo, W. Computational Screening of Bimetallic Catalysts: Application to Ammonia Decomposition. *The Journal of Physical Chemistry C* **2022**, *126* (1), 192-202.
(6) Wang, T.; Abild-Pedersen, F. Achieving industrial ammonia synthesis rates at near-ambient conditions through modified scaling relations on a confined dual site. *Proceedings of the National Academy of Sciences* **2021**, *118* (30), e2106527118.
(7) Lundberg, S. M.; Lee, S.-I. A unified approach to interpreting model predictions. *Advances in neural information processing systems* **2017**, *30*.
(8) Ho, J.; Jain, A.; Abbeel, P. Denoising diffusion probabilistic models. *Advances in neural information processing systems* **2020**, *33*, 6840-6851.
